# Breath Stable Isotope Analysis Serves as a Non-invasive Analytical Tool to Demonstrate Dietary Changes in Adolescent Students Over Time

**DOI:** 10.3389/fmed.2021.697557

**Published:** 2022-01-25

**Authors:** Christy J. Mancuso, Collette M. Cornwall, Swede Robinson, Luciano O. Valenzuela, James R. Ehleringer

**Affiliations:** ^1^Department of Biology, University of New Mexico, Albuquerque, NM, United States; ^2^School of Biological Sciences, University of Utah, Salt Lake City, UT, United States; ^3^Highland High School, Salt Lake City School District, Salt Lake City, UT, United States; ^4^Consejo Nacional de Investigaciones Científicas y Técnicas, Facultad de Ciencias Sociales, Universidad Nacional del Centro de la Provincia de Buenos Aires, Unidad de Enseñanza Universitaria Quequén, Quequén, Argentina

**Keywords:** stable isotopes, diet, exhaled breath CO_2_, high school students, Healthy Hunger-Free Kids Act (HHFKA)

## Abstract

Concern about adolescent diets, obesity, and the associated health risks have been growing in the United States. This inspired former First Lady Michelle Obama to spearhead the Healthy Hunger-Free Kids Act (HHFKA), which made changes to the national school lunch program by increasing servings of whole grains, fruits, and vegetables. Our study examined the variability of student carbohydrate sources throughout the day and before and after the implementation of HHFKA using a stable isotope dietary biomarker. This method uses carbon stable isotope values of exhaled CO_2_ breath (δ^13^C_breath_) and provides a quantitative, non-invasive measure. δ^13^C_breath_ samples were collected throughout the day from students (*n* = 31) that attended a public high school in Salt Lake City, UT. δ^13^C_breath_ measurements reflected the short-term carbohydrate inputs from the previous meal. Carbohydrate sources were not consistent throughout the day; most students had their lowest inputs of corn/sugar-based carbohydrates after lunch. We compared our results with an earlier study that had been conducted pre-HHFKA. After-lunch δ^13^C_breath_ values decreased significantly between the two time points, suggesting an increase in whole grain, fruit, and vegetable carbohydrates in the lunch program. Our results demonstrated that δ^13^C_breath_ measurements provide a valuable tool to examine carbohydrate sources in an individual's diet throughout the day. We believe that this tool could be beneficial to studies examining the relationship between sugar sweetened beverages, added sugars, and refined carbohydrates and health outcomes like diabetes and obesity in both adolescent and adult populations.

## Introduction

Childhood obesity has been a growing concern in the United States and over the past 30 years has quadrupled among adolescents (1). Studies have shown that diets rich in carbohydrates and refined sugars have contributed in the rise in obesity and cardiovascular disease (2–5). Having precise dietary measurements are critical to these studies and most tools for dietary assessments rely on self-reporting and recall of food items and quantities consumed. These assessment tools are widely used in nutrition and diet studies and have been successfully applied in understanding habitual diets in large populations; however, they require literate populations that can accurately recall the types and quantities of foods consumed, may have high participant burdens, and can be resource intense (6–10). Having a quantitative and objective biomarker of diet would be an asset to these dietary assessments and recall methodologies. Here we describe the use of a stable isotope dietary biomarker that is non-invasive, cost effective, and provides quantitative outputs that are free of reporting biases related to gender, age, or ethnicity.

Stable isotope biomarkers have readily been used to learn about diets in many disciplines ranging from animal ecology to anthropology (11–15). They are also gaining credence and popularity among nutrition and health research (10, 16, 17). In particular, carbon (δ^13^C) isotope ratios have been used because they display predictable and natural patterns that are linked to protein, carbohydrate, and lipid consumption (18, 19). Carbon in food is derived from atmospheric CO_2_ that is fixed in organic molecules through the process of photosynthesis in plants. Differences in plant photosynthetic processes (C_3_ or C_4_) generates most of the variation that is found in our food (20) and has allowed us to differentiate diets among populations (18, 21). These processes impart unique non-overlapping δ^13^C values for C_3_ (vegetables, grains, fruits; C_3_: −20 to −30‰,) and C_4_ (corn, sorghum, sugarcane; C_4_: −16 to −10‰) foods and relate to the dietary proportions of these plants that have been consumed directly by an individual or indirectly as animal feed (19–22).

In the United States corn, sugar cane, and their respective derivatives (ex. high fructose corn syrup) are some of the most wildly consumed C_4_ food products. They are integrated into human tissues through the consumption of refined carbohydrates (ex. candies, cookies), sweetened beverages, and livestock that are fed corn-based feed (23–25). Distinct differences in the δ^13^C values of these C_4_ sweetened foods allows them to be identified from C_3_ carbohydrate sources (25). While C_4_ food products (corn, millet, sorghum) are not necessarily unhealthy, the byproducts or derivatives are added to beverages and foods have been associated with increased caloric intake, weight gain, and sugar consumption that can have deleterious health outcomes (9, 23, 26). These findings have sparked the interest of medical researchers, particularly the analysis of δ^13^C values to examine elevated sugar intake in youth and adolescent populations. Many of these studies have relied on blood samples collected from a routine fingerstick. While these collection methods are minimally invasive, they still inflict discomfort to the study subject and increase potential exposure to blood borne pathogens to research personnel (9, 10, 23). Here we propose the use of exhaled breath CO_2_ δ^13^C (here forward δ^13^C_breath_) measurements, as another quantitative biomarker to examine dietary changes that would be pain free, non-invasive, and reduced risk to pathogens.

There has a been a long history of δ^13^C_breath_ analyses. Early studies were interested in understanding the baseline variation in breath δ^13^C for metabolic labeling studies (27). Recent, analytical advancements have sparked a renewed interest in δ^13^C_breath_ measurements for metabolic and dietary studies (16, 28–31). The analysis of δ^13^C_breath_ has been of interest to examine carbohydrate intake over short time scales (hours) and may reflect foods that have been immediately eaten (carbohydrates and simple sugars) or stored reserves (lipids) (31, 32). Recently, O'Brien et al. examined using δ^13^C_breath_ as a marker of short term added sugar intake in adult participants in a controlled setting (16).

Our interest in the analysis of δ^13^C_breath_ measurements in adolescent high schoolers were fueled by the growing concern of excess sweeteners in their diets, the deleterious health outcomes associated with obesity, and the implementation of the Healthy Hunger-Free-Kids Act (HHFKA) in 2012. This program was spearheaded by former First Lady Michelle Obama and promoted increased servings of whole grains, fruits, and vegetables in the national school lunch program (33). The goal of our study was to analyze δ^13^C_breath_ from high school students in Salt Lake City, UT to examine (a) broad carbohydrate sources of adolescent students throughout the day, and (b) to compare the findings of our 2016 adolescent population to that of Valenzuela et al.'s 2009–2010 population to examine if the δ^13^C_breath_ in high school students in the Salt Lake City school district have changed in conjunction with the implementation of the HHFKA.

## Methods

Thirty-one student volunteers were recruited from a public high school (grades 10–11) in Salt Lake City, Utah in 2016. Breath samples, demographic information (sex and participation in school lunch), and an anonymous diet survey were collected from volunteers. The University of Utah's Institutional Review Board approved this study (IRB 00035524) and parents/legal guardians and students provided written consent to participate and could choose which sample(s) and demographic information they provided. Hence, the sample sizes of different variables. To draw comparisons to our study population we examined a dataset of δ^13^C_breath_ samples from students in a different public high school in the Salt Lake City school district (grade 10, *n* = 33) (University of Utah's Institutional Review Board approved, IRB 00032797) that were collected in 2009–2010 and were part of a larger study from Valenzuela et al. (31).

### Breath Samples

In our study three breath samples were requested from each volunteer to measure after breakfast (AB), after lunch (AL), and after dinner (AD) δ^13^C_breath_. Students were directed to take breath measurements in a 1-to 2-h window following their meal. Volunteers were provided with straws and labeled Exetainer tubes (Labco, United Kingdom) and were instructed to inhale atmospheric air and exhale air through the coffee straw into the tube for 1 min and immediately cap the sample and return it to school the next day. AB and AD samples were collected at home and AL samples were collected at the school in a 1-to 2-hour window following lunch. All samples were collected and stored at room temperature during the school week (Tuesday – Thursday) to ensure that breath samples could be collected by teachers and delivered to the University of Utah within a 24–48 h collection window to be analyzed. The majority of the AL and AD samples from each volunteer were sampled on the same day; however, this was not always possible (i.e., students would forget to sample AD breath and sample would be taken the following evening). AB samples were collected on a different day. Although breath samples were taken on different days, we assumed that overall food choices during this time would remain consistent. Anonymous survey responses from participating students allowed us to understand habitual eating habits of the study group (Supplementary Table 1) and based upon their responses we felt that broad carbohydrate choice was consistent over the sampling period. Additionally, previous work examining breath stable isotopes found a significant covariation between breath isotopes and food based questionnaires of similarly aged students, suggesting that there may be temporal stability in the consumption of carbohydrate sources (31).

Breath samples from volunteers were analyzed at the Stable Isotope Ratio Facility for Environmental Research (SIRFER) at the University of Utah within 24–48 h of collection. Breath samples were measured using an autosampler connected to an isotope ratio mass spectrometer (Finnigan Delta Plus IRMS) via Thermo Finnigan Gasbench II (Thermo Fisher, Bremen, Germany). Breath samples and internal standards, which had previously been characterized relative to an international ^13^C standard, were analyzed in triplicate. δ^13^C measurements of AB, AL, and AD breath samples were analyzed alongside three in-house CO_2_ reference gases with isotope values of +16.2, −6.0, and −42.6‰. The analytical precision (1σ), based on repeated measurements of these references, was 0.16‰. Results for δ^13^C values are presented on the Vienna Pee Dee Belemnite scale. Stable isotope ratios are reported using the standard δ-notation relative to an international standard in units per mil (‰) using the following: δX = (R_sample_/R_standard_ – 1) ^*^ 1000, where X is the isotope of interest, R_sample_ and R_standard_ are the molar ratios of the heavy to the light isotopes (e.g.,^13^C/^12^C) of the sample and international standard, respectively.

We compared our δ^13^C_breath_ samples with that of the high school population described in Valenzuela et al. (31). In brief, they collected two breath samples from each participant, one in the morning (AM) before breakfast and an AL breath sample that was collected in a 1-to-2-h window following lunch. Samples were collected in foil balloons and analyzed on the same day at the SIRFER facility (34). Six hundred microliters of exhaled breath samples were removed from balloons using a gas tight syringe with a pressure locking valve (Pressure-Lok, Baton Rouge, Louisiana) and injected onto a gas chromatography column coupled to a Finnigan Delta Plus IRMS (Thermo Fisher, Bremen, Germany) operating in continuous flow mode. Similar to our study breath samples were analyzed alongside in-house CO_2_ reference gases with isotope values of +15.5 and −10.0‰. The analytical precision (1σ), based on repeated measurements of these references, was 0.15‰ (31).

### Statistics

Statistical significance and graphic output were generated using Prism v9.0 (GraphPad Software). Data were summarized as mean ± standard deviation (SD). Normality of distributions were examined using the Shapiro-Wilks test. Exhaled breath samples throughout the day from all participants were analyzed using an Ordinary one-way ANOVA as we had unequal number of samples at each time point. Sex and school lunch participation comparisons were analyzed using an unpaired *t*-test. To examine the relationship between δ^13^C values of exhaled breath CO_2_ after each meal Pearson correlations were made from AB vs. AL, AL vs. AD, and AD vs. AB. Comparisons between δ^13^C exhaled breath samples pre- and post- HHFKA were assessed using an unpaired *t*-test test. Results were considered significant at *p* < 0.05.

## Results

Thirty-one high school students (grades 10 and 11) in the Salt Lake City school district participated in this study (Table 1); 18 were male and 13 were female; 51% of the students reported that they participated in the school lunch program. While we did not record student ethnicity, the high school that we worked with had a predominately non-Hispanic White (49%) and Hispanic (21%) student population at time of sample collection (35). We analyzed δ^13^C_breath_ samples measurements AB (*n* = 31), AL (*n* = 24), and AD (*n* = 22). Breath sample numbers varied, as volunteers were not required to participate in every collection and could choose to leave the study at any time. Breath isotope samples were normally distributed (Shapiro-Wilk test, *P* > 0.5 for all variables, AB δ^13^C_breath_ W = 0.97, *P* = 0.62; AL δ^13^C_breath_ W = 0.95, *P* = 0.26; AD δ^13^C_breath_ W = 0.96, *P* = 0.57). Samples from our study were compared to 33 high school students from grade 10 in a different high school in the Salt Lake City school district that were collected in 2009–2010 as part of the Valenzuela et al. study (Table 1). These students were part of a larger study population that included students from grades 5–10; however, we have focused on students from grade 10, as they are most similar to our study population. From the population in the Valenzuela study, 16 participants were male and 19 were female; 63% were non-Hispanic White and 31% of the students were Hispanic. δ^13^C measurements of AM and AL (*n* = 33) exhaled CO_2_ breath samples were analyzed, and all breath samples were normally distributed (Shapiro-Wilk test, *P* > 0.5, AM δ^13^C_breath_ W =0.97, *P* = 0.62, AL δ^13^C_breath_ W = 0.97, *P* = 0.53).

**Table 1:** Summary of study population demographics.

	**2016 population**	**2009–2010 population**
**Characteristic**		
Total participants	31	33
**Sex**		
Male	18	16
Female	13	19
**Participation in school lunch**		
% Yes	51%	N/A
**Race/ethnicity**		
% Non-hispanic white	49%^*^	63%
% Hispanic	21^*^	31%

*Samples from 2009–2010 study population were collected and further described in Valenzuela et al. (31)*.

* *Race/ethnicity was not collected for 2016 study population, values listed were reported for the entire high school population (35)*.

### Variation in Breath δ^13^C Throughout the Day

**Figure 1:**
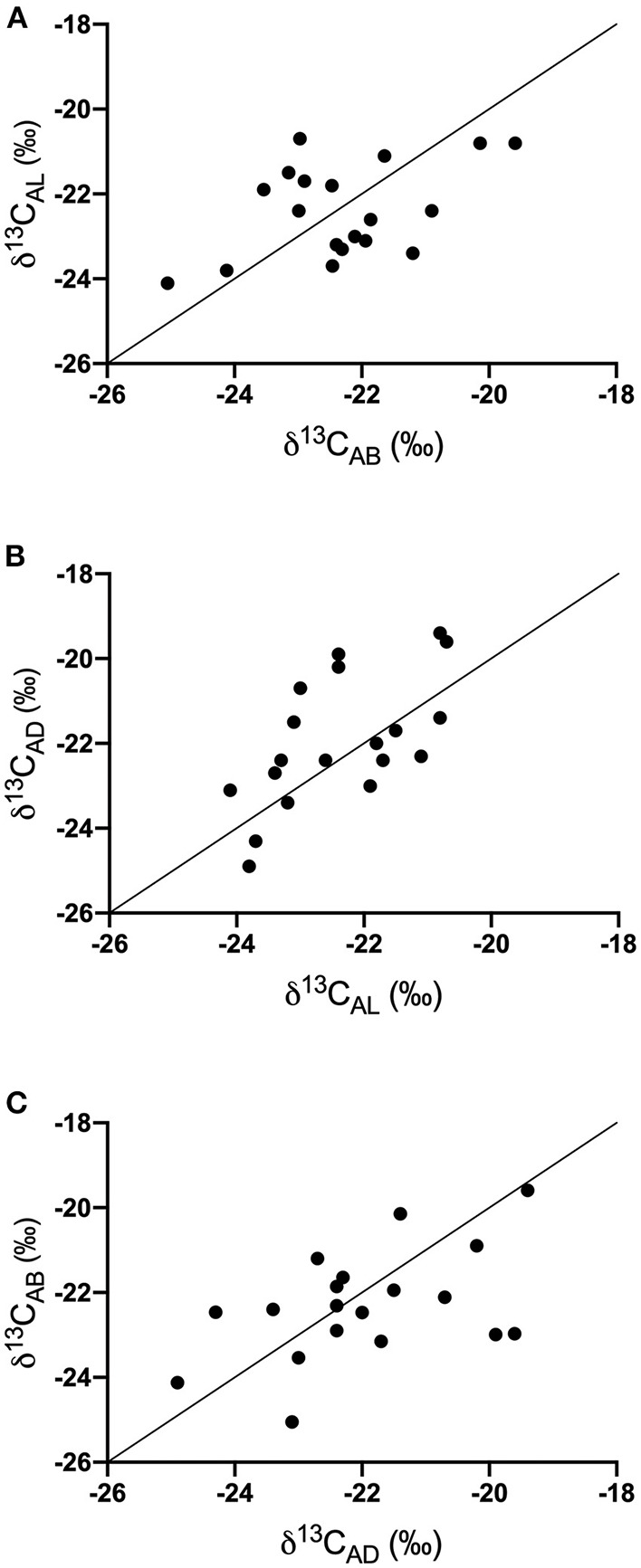
Covariation of δ^13^C values of exhaled breath samples throughout the day **(A)** AB vs. AL, **(B)** AL vs. AD, and **(C)** AD vs. AB. Solid line represents the 1:1 line.

δ^13^C_breath_ values (Supplementary Table 2) were not significantly different among demographic variables (sex and school lunch participation) or across the different time points (AB, AL, or AD) for the entire study population (*n* = 31). The variation in sample number for each time point could have contributed to these findings. To ensure that an equal number of samples were present at each time point, we examined samples only from study subjects that contributed breath samples at all time points (*n* = 19) for the following analyses. Breath samples were not always collected over sequential days, but we made the assumption that dietary choices were similar on a day-to-day basis based on anonymous survey responses from students, interactions with teachers, and previous studies (31). We compared the change in δ^13^C_breath_ after each meal and found there were positive correlations between all analyses (Figure 1), with significant relationships for analyses comparing AL vs. AD (*p* = 0.004, Pearson *r* = 0.631) and AD vs. AB (*p* = 0.050, Pearson *r* = 0.456). We did not find significant correlations between AB vs. AL (*p* = 0.055, Pearson *r* = 0.447). Examination of the difference between the after-meal breath isotope values, relative to the earlier meal revealed that at the individual level few volunteers maintained a constant isotope value throughout the day. Fifty three percent of AL δ^13^C_breath_ samples were more negative relative to AB (Figure 1A). Forty-seven percent of AL δ^13^C_breath_ samples were more negative relative to AD, while 21% remained consistent between AL and AD samples (Figure 1B). These findings suggest that for most of the study subjects lunch had the most negative δ^13^C_breath_ values and would be consistent with greater proportions of C_3_ foods.

### Comparison of δ^13^C Breath Isotopes Pre- and Post-HHFKA

**Figure 2:**
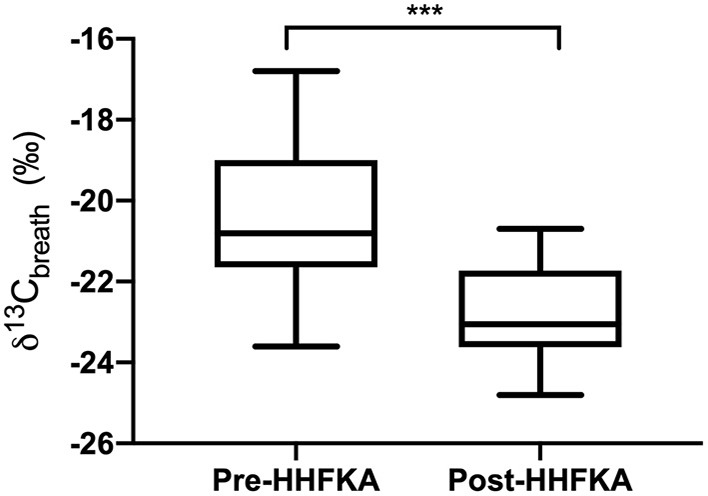
After lunch carbon isotope values of exhaled breath display differences pre- and post HHFKA. Box and whiskers plot of carbon isotope values of exhaled breath CO_2_ collected after lunch from high school students (grades 10-11) before the Healthy, Hunger Free Kids Act (31); Pre-HHFKA, *n* = 33 and after Post-HHFKA, *n* = 24. ^***^p < 0.001, unpaired *t*-test.

Lastly, we compared our AL δ^13^C_breath_ values with samples that were collected as part of the Valenzuela et al. study to examine changes to δ^13^C_breath_ values pre- and post- HFFKA implementation. Examination of the δ^13^C_breath_ values between the two studies highlighted a shift in the AL δ^13^C_breath_ samples being more negative post-HHFKA [Figure 2, *p* < 0.001, unpaired *T*-test, pre- (*n* = 33) and post- (*n* = 24)]. While our study did not record ethnicity from study subjects, 31% of the breath samples from the Valenzuela study came from Hispanic students, where they noted a significant elevation in sweetened beverage servings per week compared to the non-Hispanic White students across all participants in their study (31). The high school that our students attended had a 21% Hispanic student population. To potentially mitigate against the C_4_ bias that could exist in the Hispanic population from the Valenzuela study, we also compared the δ^13^C_breath_ samples from only the non-Hispanic White students (*n* = 22) to our study subjects (*n* = 24) and we still saw that δ^13^C_breath_ samples were more negative post-HHFKA (*p* = 0.0009, unpaired *t*-test). We also compared students in our population that ate school lunch (*n* =12) and the non-Hispanic White students (*n* = 22) in the Valenzuela study and δ^13^C_breath_ samples were still more negative post-HHFKA (*p* = 0.0026, unpaired *t*-test). Lastly, we examined differences between the AM breath samples collected from Valenzuela et al. to our other time points and found that there were statistical differences between the two studies (AM vs. AB, *p* = 0.0003, unpaired *t*-test and AM vs. AD, *p* = 0.0038, unpaired *t*-test).

## Discussion

This study has demonstrated that δ^13^C_breath_ measurements can be used as a valuable non-invasive tool to examine recent carbohydrate consumption in an individual's diet throughout the day. Our study focused on samples from students after each meal to look at the variability in δ^13^C_breath_ values throughout the day. We found that δ^13^C_breath_ samples broadly quantified dietary inputs from carbohydrate sources over a period of hours. When we examined the δ^13^C_breath_ samples throughout the day among all of our study participants (*n* = 31), we did not find significant differences in the δ^13^C_breath_ values across the collection time points (Supplementary Table 2). Our results could support two conclusions (1) that as a population our students consumed similar carbohydrate sources throughout the day or (2) that previous meals could be influencing the δ^13^C_breath_ values at the different time points. There has been evidence that δ^13^C_breath_ measurements are representative of the most recent carbohydrate sources that an individual has consumed (23), but how recent may depend on isotopic turnover and the use of internal sources (32).

Studies from Scholler et al. and O'Brien et al. have provided evidence for the rapid utilization of carbohydrates in δ^13^C_breath_ measurements. Scholler et al., found that exhaled breath CO_2_ in infants being fed a glucose-amino acid mixture immediately began oxidizing carbohydrate sources and that their average δ^13^C values were similar to that of the glucose solution (27). Similarly, O'Brien et al. found that δ^13^C_breath_ in healthy adults fed a diet of increased added sugar displayed changes in their δ^13^C_breath_ within 2 h of a meal. They also examined δ^13^C_breath_ samples from healthy adult participants (*n* = 3) every 10 min following a meal high in added sugar. δ^13^C_breath_ samples rapidly increased over the first hour, remained steady for 2 h, and declined an hour before lunch (16). While both studies agree that immediate δ^13^C_breath_ measurements reflect recently consumed carbohydrates and simple sugars, their findings differ on the amount of time that a previous meal could influence the subsequent meal δ^13^C_breath_ values. Scholler found that labeled glucose and sugars peaked in breath ^13^CO_2_ excretion around 4 h and remain in the breath for up to 8 h (27). While the O'Brien study found that following a meal δ^13^C_breath_ peaked after 2 h and decreased 4 h after the meal; however δ^13^C_breath_ 4 h post meal did not return to the pre-feeding values, which could suggest some carry over to the next meal (16).

It is likely that our results from our entire study population are a mix of both immediate utilization of carbohydrates from the most recent meal and potentially some carry over from the prior meal. Both studies showed there was rapid incorporation of carbohydrates from the consumed meal that peaked within 1–2 h, like our breath collection time points. It is possible that residual carbon from the previous meal could be present in our AL or AD samples and contribute to the similarity in the δ^13^C_breath_ values of our entire study population. However, it seems that our samples are more like O'Brien et al., as both study subjects consumed dietary foods opposed to glucose solutions. While we cannot exclude the potential of some residual carbon from the previous meal it seems unlikely to have had a major impact on the δ^13^C_breath_ value since meal breath samples were taken well-after the 4-h post-meal time point (16). Additionally, δ^13^C_breath_ values at the individual level support the O'Brien study's time period as we saw that among individual students δ^13^C_breath_ values changed throughout the day. Specifically, that AL δ^13^C_breath_ samples relative to either AB or AD meals suggested that that more C_3_ carbohydrate sources were consumed at lunch.

To examine the potential changes associated with the implementation of HHFKA, we compared the δ^13^C_breath_ samples (post-HHFKA) from our population (2016) to the high school population described in Valenzuela et al.'s 2009–2010 study (pre-HHFKA) (31). δ^13^C_breath_ samples pre-HHFKA were significantly higher and suggest that students consumed more C_4_ processed carbohydrates that had added sugars (i.e., corn or cane sugars) during their lunch compared to observations in our study. It is possible that the δ^13^C_breath_ values of the pre-HHFKA population was influenced by food choices from the Hispanic students in the Valenzuela study, which comprised 31% of the analyzed samples listed here. Valenzuela et al. reported that Hispanic students, in their entire study, had significantly higher servings per week of sweetened beverages compared to non-Hispanic White students (31).

Since we did not record the ethnicities of our study population and we know that the overall high school student population was non-Hispanic White (49%) we wanted to try to mitigate the potential effect of differences in ethnic groups represented in the Valenzuela study (35). When we compared δ^13^C_breath_ samples from only the non-Hispanic White students in the Valenzuela study to our study, we still saw that there was a significant difference between the two time periods. These results suggest that our study population were incorporating greater contributions of C_3_ carbohydrates (fruits, vegetables, and whole grain sources) that are consistent with the changes outlined in the HHFKA. Additionally, our findings are supported by Liu et al.'s most recent paper that examined dietary surveys from 2003–2004 and 2017–2018 among 20,905 school age children (ages 5–19 years old) and found that children consuming food with poor diet quality from school meals decreased by half [55.6% (2003–2004) to 24.4% (2017–2018)] and were attributed to increases in whole grains and fruits and decreased sugar sweetened beverages (36) consistent with the changes outlined in HHFKA.

Our comparison of δ^13^C_breath_ is one of the first studies to examine the use of a non-invasive biomarker to assess broad types of foods that students consumed before and after the implementation of the HHKFA. We acknowledge that our study assessed a small population of high school aged students compared to other diet studies and had some limitations (student food recalls, ethnic demographic information was not taken, and the same students from the Valenzuela study were not enrolled). Despite these limitations, we have matched our comparison group to the best of our abilities (high school aged students, within the same public school district, similar high school ethnic demographics) and our findings can be considered more preliminary in the context of policy implementation; however, our work highlights a useful technique that can assess changes in dietary carbohydrate sources. Specifically, our study highlights the C_4_-to C_3_ change in carbohydrate sources before and after HHFKA and is consistent with the literature supporting the implementation of act. Specifically, the studies that have shown that students are consuming greater proportions of fruits, vegetables, and whole grains in their entrées (36–38), after HHFKA, contributing to the overall positive impact that the program has had. It is interesting to note that we also saw significant differences between the δ^13^C_breath_ values in the AD and AB samples compared to the Valenzuela et al.'s AM beath samples. Perhaps these findings represent an overall change in diet and food selection preferences from 2009–2010 to 2016 or perhaps, that lunch represents a larger proportional input to breath samples later in the day. However, we have no data, nor have there been specific published studies that would allow us to test this specific hypothesis, but we feel this is an interesting question that deserves further consideration.

## Conclusions

This study has shown that δ^13^C measurements of breath samples can provide insight into the food choices and diets of American high school adolescents. We observed that school lunch can have an impact on a student's diet and that AL δ^13^C_breath_ samples have significantly changed after the implementation of HHFKA. While stable isotope analyses of breath samples are useful in understanding an individual's diet, there are application limitations. Stable isotopes provide us with general information related to the broad characterization of different food types or sources. δ^13^C measurements allow us to distinguish food groups based on photosynthetic pathways; however, they do not allow us to differentiate an apple from an orange, both C_3_ food sources. These broad distinctions may have more local applications in the Americas, as beet sugars would have similar δ^13^C values to other C_3_ food sources. Isotopes alone offer broad dietary information and in combination with dietary recall methodologies could provide specificity to those isotope values while also confirming food choices on the questionnaires.

We would encourage researchers to continue to explore the use of stable isotope analyses of human samples, especially fingernail clippings, hair samples, and saliva as they can be collected non-invasively from study subjects and reflect short and long term dietary inputs. Compound specific isotope analyses of these tissues is another area that should also be examined, as δ^13^C measurements of individual amino acids may provide additional insight and specificity to broad characterization of food types (39). While our study focused on adolescent populations and the role of the school lunch program, we believe that this application may have significant benefits to childhood obesity and diabetic medical studies.

## Data Availability Statement

The raw data supporting the conclusions of this article will be made available by the authors, without undue reservation.

## Ethics Statement

The studies involving human participants were reviewed and approved by University of Utah's Institutional Review Board. Written informed consent to participate in this study was provided by the participants' legal guardian/next of kin.

## Author Contributions

CM, SR, CC, and JE designed the research. CM, SR, and CC conducted the research. CM analyzed the data and wrote the article. LV and JE helped with comments in the writing of article. CM, LV, and JE had primary responsibility for final content. All authors contributed to the article and approved the submitted version.

## Funding

Funding for CM during manuscript preparation was supported by funding from the National Institute of General Medical Sciences K12 GM08821.

## Conflict of Interest

The authors declare that the research was conducted in the absence of any commercial or financial relationships that could be construed as a potential conflict of interest.

## Publisher's Note

All claims expressed in this article are solely those of the authors and do not necessarily represent those of their affiliated organizations, or those of the publisher, the editors and the reviewers. Any product that may be evaluated in this article, or claim that may be made by its manufacturer, is not guaranteed or endorsed by the publisher.
