# Supplementary material for: Breath Stable Isotope Analysis Serves as a Non-invasive Analytical Tool to Demonstrate Dietary Changes in Adolescent Students Over Time
Source: Front Med (Lausanne). 2022 Jan 25;8:697557. doi: 10.3389/fmed.2021.697557 (PMC8821816; doi:10.3389/fmed.2021.697557)
Supplement: Supplementary file 1 [file Table_1.pdf]

Supplementary Material

1 Supplementary Tables

S.Table 1. Summary of anonymous food survey given to grade 10 and 11 students in 2016 population to understand dietary habits and food choices.

**General Diet Habits**		**% Student Responses**
1	Typically, which meals do you eat at school?	
	None	39%
	Breakfast	2%
	Lunch	39%
	Breakfast and Lunch	20%
2	Which of the following best describes your dietary habits	
	Vegetarian	4%
	Vegan	0%
	I eat a little meat	17%
	I eat meat often	79%
3	Which of the following meats do you eat regularly	
	Chicken	40%
	Beef	30%
	Pork	17%
	Fish	12%
	Seafood	1%
**Breakfast Habits**		
1	Which of the following describes your breakfast habits	
	I often skip breakfast	34%

	I will most likely grab something from home on the go	21%
	I will sit at home and eat	27%
	I will get fast food	2%
	I will eat breakfast from the school	16%
2	What carbohydrates do you typically eat for breakfast?	
	Cereal/Granola Bar	61%
	Pancake/Waffle	14%
	Oatmeal	4%
	Toast	18%
	Pop Tarts	3%
3	What proteins do you typically eat for breakfast?	
	Eggs	31%
	Bacon, Sausage, Meat	21%
	Protein bar/shake	18%
	Yogurt	9%
	I don’t eat these things	20%
4	What types of fruit do you typically eat for breakfast?	
	Canned fruit	1%
	Fruit cup	12%
	Fresh fruit	54%
	Dried fruit	2%
	I don’t eat fruit often	31%
5	With my breakfast I will typically drink	
	Milk	56%
	Whole fruit juice	29%
	Tomato juice	3%
	Sunny D	10%
	Soda	3%
6	If eating breakfast at school in the morning I would choose	

	Breakfast pizza	30%
	Yogurt parfait	17%
	Muffin	17%
	Cereal	21%
	Pancake on a stick with sausage	15%
7	If eating breakfast at school, I would choose the following sides	
	Hash browns	58%
	Strawberry Slices	14%
	Peaches, pears, or mixed fruit	16%
	Apple Sauce	9%
	Ketchup/Salsa	5%
**Lunch Habits**		
1	Which of the following best describes your typical lunch	
	I will bring lunch from home	17%
	I will eat school lunch	45%
	I will go off campus to a restaurant	11%
	I will go home for lunch	10%
	I often skip lunch	17%
2	Which lunch side would you choose	
	Sweet potato fries	11%
	Carrots	13%
	Red grapes	33%
	Mashed potatoes	39%
	Corn salad	4%
3	If I want a snack, I will typically get	
	Candy bar	16%
	Corn chips	26%
	Potato chip	19%
	Fresh fruit	26%
	Crackers/cookies	14%

4	Typically at lunch I will drink	
	Water	51%
	Soda	14%
	Diet Soda	5%
	Fruit juice	16%
	Sport drinks	15%
**Dinner Habits**		
1	How often do you have meat with your dinner	
	Never	5%
	Sometimes	33%
	Often	62%
2	Which veg do you often eat with dinner	
	Corn	16%
	Mixed Veg	29%
	Broccoli	15%
	Bean	14%
	Salad	27%

S.Table 2. Summary Data of 2016 Population**.** Carbon isotopes of exhaled breath CO_2 after meals. Statistical summary of exhaled carbon isotope values of breath after breakfast, lunch, and dinner. An unpaired t-test was used to make between variable comparisons (sex and school lunch participation) at each time point, and determination of changes throughout the day were made using an Ordinary one-way ANOVA. Isotope values reflect mean ±SD

**Breath Isotopes**	**After Breakfast**			**After Lunch**			**After Dinner**			**Throughout Day**
**Variable**	**N**	**$\delta^{13}C \pm SD$ (‰)**	**p-value**	**N**	**$\delta^{13}C \pm SD$ (‰)**	**p-value**	**N**	**$\delta^{13}C \pm SD$ (‰)**	**p-value**	**p-value**
**Mean**	31	-22.3 ± 1.30		24	-22.7 ± 1.20		22	-22.1 ± 1.50		0.3609
**Sex**										
Male	18	-22.0 ± 1.60	0.11	14	-22.8 ± 1.30	0.6139	12	-22.3 ± 1.04	0.6312	0.2337
Female	13	-22.7 ± 0.81		10	-22.6 ± 1.03		10	-22.0 ± 1.6		0.4221
**School Lunch**										
Yes	16	-22.3 ± 0.99	0.8610	12	-22.7 ± 1.20	0.8198	11	-21.7 ± 1.30	0.1674	0.1938
No	15	-22.2 ± 1.70		12	-22.7 ± 1.10		11	-22.3 ± 1.30		0.3838
